# DNA methylation and healthy human aging

**DOI:** 10.1111/acel.12349

**Published:** 2015-04-25

**Authors:** Meaghan J. Jones, Sarah J. Goodman, Michael S. Kobor

**Affiliations:** ^1^ Centre for Molecular Medicine and TherapeuticsChild and Family Research InstituteUniversity of British ColumbiaVancouverBCCanada; ^2^ Department of Medical GeneticsUniversity of British ColumbiaVancouverBCCanada; ^3^ Human Early Learning PartnershipSchool of Population and Public HealthUniversity of British ColumbiaVancouverBCCanada

**Keywords:** aging, DNA methylation, epigenetics, human, review

## Abstract

The process of aging results in a host of changes at the cellular and molecular levels, which include senescence, telomere shortening, and changes in gene expression. Epigenetic patterns also change over the lifespan, suggesting that epigenetic changes may constitute an important component of the aging process. The epigenetic mark that has been most highly studied is DNA methylation, the presence of methyl groups at CpG dinucleotides. These dinucleotides are often located near gene promoters and associate with gene expression levels. Early studies indicated that global levels of DNA methylation increase over the first few years of life and then decrease beginning in late adulthood. Recently, with the advent of microarray and next‐generation sequencing technologies, increases in variability of DNA methylation with age have been observed, and a number of site‐specific patterns have been identified. It has also been shown that certain CpG sites are highly associated with age, to the extent that prediction models using a small number of these sites can accurately predict the chronological age of the donor. Together, these observations point to the existence of two phenomena that both contribute to age‐related DNA methylation changes: epigenetic drift and the epigenetic clock. In this review, we focus on healthy human aging throughout the lifetime and discuss the dynamics of DNA methylation as well as how interactions between the genome, environment, and the epigenome influence aging rates. We also discuss the impact of determining ‘epigenetic age’ for human health and outline some important caveats to existing and future studies.

## Introduction

The biology of aging has been a consistent source of interest among the public and researchers alike. Main questions for exploration include what happens to result in the host of phenomena associated with age and what strategies can slow this progression. Beginning at birth, developmental programs result in age‐related changes to gene expression, growth, and physiology. Over the entire life course, these changes can be seen in physical manifestations, especially in advanced age with its associated functional declines caused by the accumulation of cellular damage, paired with a diminished ability to repair this damage (Lopez‐Otin *et al*., 2013).

One phenomenon associated with aging is a change in patterns of epigenetic modifications. Epigenetics is most commonly defined as modifications to DNA and DNA packaging that do not involve changes to the DNA sequence and that are potentially transmissible to daughter cells (Bird, 2007). Given its dynamic nature, epigenetics has been referred to as the interface between the genome and the environment (Feil & Fraga, 2012). Epigenetics includes modifications to histone proteins, noncoding RNAs, and DNA methylation; here, we focus on the latter as it is a more accessible mark for quantitative measurements and thus is more often used in human population studies. The most common form of DNA methylation involves the addition of a methyl group to the 5′ cytosine of C‐G dinucleotides, referred to as CpGs. These nucleotide pairs are relatively sparse in the genome, and areas of comparatively high CpG density are referred to as CpG islands, identified as regions > 200 bp with a > 50% G+C content and 0.6 observed/expected ratio of CpGs (Saxonov *et al*., 2006; Illingworth & Bird, 2009). These islands tend to be less methylated compared to nonisland CpGs and are often associated with gene promoters, while the regions immediately surrounding CpG islands are referred to as ‘shores’, followed by ‘shelves’. Approximately 60–70% of genes have a CpG island associated with their promoters, and promoters can be classified according to their CpG density (Saxonov *et al*., 2006; Weber *et al*., 2007). Levels of DNA methylation at a promoter‐associated CpG island are generally negatively associated with gene expression, although some specific genes show the opposite effect (Weber *et al*., 2007; Lam *et al*., 2012; Gutierrez Arcelus *et al*., 2013). Interestingly, this negative correlation is not upheld when comparing expression and DNA methylation for a specific gene across individuals (van Eijk *et al*., 2012; Lam *et al*., 2012; Gutierrez Arcelus *et al*., 2013; Wagner *et al*., 2014). Conversely, DNA methylation in the gene body is often positively associated with levels of gene expression (Lister *et al*., 2009; Gutierrez Arcelus *et al*., 2013). DNA methylation also functions to repress repetitive elements, such as Alu and LINE‐1, which are generally highly methylated in the human genome. (Kochanek *et al*., 1993; Alves *et al*., 1996).

Just as patterns of gene expression differ across tissues, so do patterns of DNA methylation (Byun *et al*., 2009; Ziller *et al*., 2013). In fact, tissue of origin is the primary difference in DNA methylation profiles from different samples, regardless of whether they originate from the same or different individuals (Davies *et al*., 2012; Ziller *et al*., 2013; Jiang *et al*., 2015). DNA methylation can affect transcription factor binding sites, insulator elements, and chromatin conformation, resulting in multiple levels of control of expression (reviewed in (Jones, 2012)). In addition to normal DNA methylation, variations and derivatives of the methyl mark on CpG dinucleotides have also been reported, including hydroxymethyl, formyl, and carboxyl groups, as well as methyl marks that occur at non‐CpG sites. These variants are very rare and many of the technologies commonly used to assess DNA methylation do not distinguish between them, and so for the purposes of this review, all such modifications to DNA will be referred to as ‘DNA methylation’.

Recently, there has been an increase in the number of studies examining associations between DNA methylation in age across the lifespan and across tissues. In this review, we outline the dynamics of DNA methylation over the lifespan and define two distinct phenomena that collectively contribute to these changes. We focus only on healthy aging; for excellent reviews of the epigenetics of age‐associated disease including cancer, please see Baylin & Jones (2011), Bergman & Cedar (2013), and Teschendorff *et al*. (2013).

## DNA methylation dynamics during aging

Changes in DNA methylation occur throughout the lifetime, beginning at conception. Early studies assessing levels of DNA methylation both globally and at specific regions observed age‐associated changes (Wilson *et al*., 1987; Drinkwater *et al*., 1989; Fuke *et al*., 2004; Kwabi‐Addo *et al*., 2007). These studies examined DNA methylation through assessment of global methylcytosine/cytosine ratios by immune, colorimetric, and HPLC analyses, and occasionally by assaying DNA methylation at repetitive elements. Based on these early studies, it was hypothesized that DNA methylation was not accurately maintained over cell divisions, resulting in a gradual loss and increase in variability over the lifespan (Cooney, 1993). This phenomenon has been referred to as ‘epigenetic drift’ (Egger *et al*., 2004; Martin, 2005). More recent work has shown that both Alu and LINE‐1 repetitive elements exhibit decreased DNA methylation levels and increased variability with age (Bollati *et al*., 2009; Talens *et al*., 2012). Furthermore, samples from the same individuals taken 8 years later showed that Alu elements lose methylation longitudinally, further verifying this pattern (Bollati *et al*., 2009). Together, these data demonstrate that gradual change in DNA methylation occurs with age, both generally across the genome and specifically at repetitive elements.

With the advent of microarray technology, it became possible to assess a large number of specific genomic sites for age‐related changes in DNA methylation. Microarray studies confirmed a decrease in DNA methylation with age, while site‐specific analysis indicated an increase in variability of DNA methylation with age. The latter was first noted in monozygotic twins, and subsequently in unrelated individuals (Fraga *et al*., 2005; Martin, 2005; Poulsen *et al*., 2007; Kaminsky *et al*., 2009; Martino *et al*., 2011). These studies also supported the idea that DNA methylation showed reduced stringency in maintenance over the lifespan, resulting in an increase in interindividual variability along with the overall decrease in DNA methylation. Hereafter, findings discussed here are quantitative and sequence‐ or array‐based except where noted.

Recent studies have expanded our understanding of the dynamics of DNA methylation over the lifespan, identifying genomic locations where changes preferentially occur. In neonatal blood, DNA methylation levels are lower than that observed at most other points during the lifespan (Martino *et al*., 2011; Wang *et al*., 2012). Interestingly, the age of the parents appears to affect DNA methylation in a subset of sites, perhaps relating to evidence that sperm DNA methylation is also associated with age (Adkins *et al*., 2011; Jenkins *et al*., 2013). After birth, average DNA methylation levels increase in blood throughout the first year of life (Martino *et al*., 2011; Herbstman *et al*., 2013). These changes occur preferentially at CpG island shores and shelves, enhancers, and promoters lacking CpG islands (McClay *et al*., 2014). In both blood and buccal epithelial cells, DNA methylation between monozygotic twins has been shown to become more variable in the first year of life (Martino *et al*., 2011, 2013). This phenomenon may indicate that the shared prenatal environment confers a high degree of epigenetic similarity between children, whereas subsequent variations in the postnatal environment result in epigenetic divergence after birth. After the first year, median global DNA methylation levels are relatively stable, with the exception of certain regions that frequently gain DNA methylation (Martino *et al*., 2011).

The first few years of life have been extensively studied; however, there are relatively few reports of changes in DNA methylation throughout later childhood and adolescence. Studies that examined this period of human development have reported that DNA methylation levels increase rapidly and then stabilize by adulthood in both brain and blood (Alisch *et al*., 2012; Lister *et al*., 2013). From adulthood to advanced age, overall levels of DNA methylation remain stable in blood, while interindividual variability increases over that time (Talens *et al*., 2012; Weidner *et al*., 2014). Postadulthood, many studies have found a mean decrease in blood DNA methylation with increasing age (Bjornsson *et al*., 2004; Boks *et al*., 2009; Heyn *et al*., 2012; Horvath *et al*., 2012; Hannum *et al*., 2013; Johansson *et al*., 2013; Florath *et al*., 2014; Weidner *et al*., 2014). These changes are less likely to occur in promoters and more likely to be observed in enhancers (Johansson *et al*., 2013). Regions that gain DNA methylation with age are enriched for CpG islands, while nonislands tend to lose DNA methylation with age (Rakyan *et al*., 2010; Heyn *et al*., 2012; Horvath *et al*., 2012; Florath *et al*., 2014; Weidner *et al*., 2014).

These findings demonstrate an interesting pattern—sites that show overall low DNA methylation, such as promoter‐associated CpG islands, tend to increase methylation with age, while those with high DNA methylation such as intergenic nonisland CpGs tend to lose methylation with age. As most CpGs are located outside of CpG islands and are highly methylated, this translates to an overall loss of DNA methylation in later life as well as a tendency for DNA methylation levels to shift toward the mean with increased age (Saxonov *et al*., 2006; Weber *et al*., 2007; Illingworth & Bird, 2009; Hannum *et al*., 2013; Teschendorff *et al*., 2013; Weidner *et al*., 2014).

Despite a gain in DNA methylation in early life and gradual loss in later life across the genome, these changes are not symmetrical. They differ in two major ways: (i) the rate of change is much higher in early life than later life, and (ii) the genomic locations of the changes are quite different. In early life, DNA methylation is gained globally, but more at island shores and intergenic regions, while in later life, DNA methylation is lost globally, but still gained at islands and shores (Alisch *et al*., 2012; Gentilini *et al*., 2013; McClay *et al*., 2014).

Beyond the general trends of DNA methylation levels changing with age, more specific examples of age‐related DNA methylation changes can be seen. Gain of DNA methylation during aging has been reported to be enriched at targets of Polycomb proteins, which generally show high levels of DNA methylation (Viré *et al*., 2006; Teschendorff *et al*., 2010; Heyn *et al*., 2012; Horvath *et al*., 2012). Beyond Polycomb targets, we can expect future research will decipher the chromatin neighborhoods associated with aging‐related DNA methylation changes.

Although the patterns described above have been mostly observed in blood, they have been replicated in other tissues. Overall, most tissues fit with the pattern of increase in average DNA methylation early in life, with a gradual decrease later in life (Grönniger *et al*., 2010; Ong & Holbrook, 2014). For example, many studies have shown that brain regions follow this pattern, showing rapid changes in DNA methylation in the early life period and then slowing gradually over the lifespan (Horvath *et al*., 2012; Numata *et al*., 2012 Lister *et al*., 2013). Given that DNA methylation profiles are highly divergent in different tissues, comparing DNA methylation across tissues presents unique challenges.

In addition to general patterns of DNA methylation change with age, it has been repeatedly shown that DNA methylation levels at specific sites in the genome are so highly associated with age and that in some cases, they have been used to accurately predict chronological age (Bocklandt *et al*., 2011; Horvath *et al*., 2012; Hannum *et al*., 2013; Florath *et al*., 2014; Weidner *et al*., 2014). These sites underlie the concept of the ‘epigenetic clock’. While this term was originally coined to describe a multivariate age predictor, it is clear that the phenomenon of the epigenetic clock is also reflected in studies that reported highly age‐associated CpGs. These epigenetic clock sites have been found both within a specific tissue and across tissues, and have been shown to be much more concordant across tissues than gene expression changes across tissues with age (Horvath *et al*., 2012; Hannum *et al*., 2013; Horvath, 2013;Florath *et al*., 2014; Weidner *et al*., 2014). These findings imply that the epigenetic clock is distinct from age‐related epigenetic drift.

## Epigenetic drift vs. the epigenetic clock: two phenomena underlying the relationship between DNA methylation and aging

**Figure 1:**
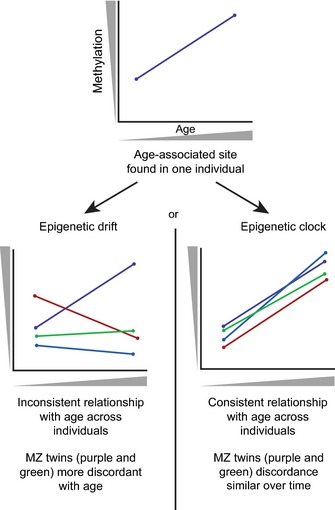
Schematic representation of epigenetic drift versus the epigenetic clock. If a specific CpG site is associated with age (top) within an individual, it may be undergoing either epigenetic drift (left) or an epigenetic clock site (right). Both phenomena have different characteristics when examined across a population or within a twin set.

Both epigenetic drift and the epigenetic clock contribute to age‐related DNA methylation changes, but in fundamentally different ways. While both are related to age, epigenetic drift represents the tendency for increasing discordance between epigenomes over time. Conversely, the epigenetic clock refers to specific sites that are consistently related to age across individuals. In some studies, the terms epigenetic drift and the epigenetic clock have been used interchangeably, though the necessity to discriminate between them has been identified (Teschendorff *et al*., 2013). Here, we define epigenetic drift as the collection of DNA methylation changes that are associated with age within an individual but are not common across individuals. The epigenetic clock, on the other hand, represents those sites that are associated with age across individuals and can thus in some cases be used to predict chronological age (Fig. 1).

Epigenetic drift is now understood to comprise age‐related changes in the epigenome that include those that are acquired environmentally as well as stochastically (Fraga *et al*., 2005; Fraga & Esteller, 2007; Kaminsky *et al*., 2009; Hannum *et al*., 2013; Teschendorff *et al*., 2013). Early indications of epigenetic drift were noted in cell culture studies, after the observation that clones of a single cell line became epigenetically divergent upon multiple passages (Humpherys *et al*., 2001). The concept was then used to describe the increase in discordance of DNA methylation between monozygotic twins as they age (Fraga *et al*., 2005; Fraga & Esteller, 2007; Poulsen *et al*., 2007). Since then, it has been shown that epigenetic drift does not necessarily occur at specific CpGs across individuals, as a site that undergoes a stochastic change in DNA methylation in one person is not likely to show the same relationship with age in another person (Fig. 1). Epigenetic drift can be observed generally in cross‐sectional studies, as evidenced by increased discordance between epigenomes with advanced age (Heyn *et al*., 2012; Talens *et al*., 2012). However, in order to identify regions that are susceptible to epigenetic drift within an individual, longitudinal studies are required.

In contrast, epigenetic clock sites show a relationship between age and DNA methylation that is consistent between individuals (Horvath *et al*., 2012; Hannum *et al*., 2013). The hypothesis behind this phenomenon rests upon the idea that specific sites in the genome undergo changes in DNA methylation with age that are progressive and common across individuals and sometimes even tissues (Horvath, 2013). Several recent studies have attempted to differentiate sites comprising the epigenetic clock from background epigenetic drift by determining which CpG sites that change with age are found across a population (Rakyan *et al*., 2010; Bocklandt *et al*., 2011; Bell *et al*., 2012; Horvath *et al*., 2012; Numata *et al*., 2012; Horvath, 2013; Florath *et al*., 2014; Weidner *et al*., 2014). Interestingly, although epigenetic age and chronological age are highly correlated across study population, there is significant interindividual variability (Horvath, 2013; Weidner *et al*., 2014). This indicates that some people's epigenome is more concordant with their chronological age than others. When tracked in a large population, the distribution of DNA methylation levels suggests that these sites change rapidly with age until adulthood, at which point the rate of change slows considerably (Horvath, 2013; Lister *et al*., 2013). An important characteristic of the epigenetic clock is that it can be tissue specific, meaning that different sites may be better correlated with age in specific tissues, although pan‐tissue epigenetic clocks have also been identified (Teschendorff *et al*., 2010; Horvath *et al*., 2012; Day *et al*., 2013). In one study, an analysis examining 20 different tissues using a multitissue‐derived age predictor found that tissues differ in their apparent epigenetic ages, with some reflecting chronological age more accurately than others (Horvath, 2013). It is likely, then, that there may exist specific sites within a tissue that more accurately predict chronological age for that tissue. Many factors of tissues or cell types may affect their apparent epigenetic aging rate, including differences in cell division rate, respiration rate and energy expenditure, or exposure to environmental factors. Interestingly, analysis of induced pluripotent stem cells shows that their predicted epigenetic age is significantly lower and very close to 0 compared to their source somatic tissues (Horvath, 2013).

### Concordance in epigenetic clock sites across studies

A number of studies have published lists of epigenetic clock sites in blood (Bocklandt *et al*., 2011; Rakyan *et al*., 2011; Bell *et al*., 2012; Numata *et al*., 2012; Hannum *et al*., 2013; Horvath, 2013; Florath *et al*., 2014; Weidner *et al*., 2014). For the purposes of this review, we attempted to identify high‐confidence candidates for epigenetic clock sites in blood based on the most highly replicated age‐related sites across eight studies. These studies were all performed using either the Illumina Infinium HumanMethylation27 or HumanMethylation450 BeadChip (27k array and 450k array, respectively) and were all performed on peripheral blood. We found no sites that replicated in seven or more studies, and decided on a list of 14 sites that were identified in a minimum of four studies. To minimize potential confounding by interindividual differences in blood cell composition (see below for more detail), we filtered for sites that were found in the only study that compared age‐associated sites to sites known to be associated with blood cell type (Weidner *et al*., 2014). This resulted in a final list of 11 CpGs, shown in Table 1. It is important to note that Study 5 identified pan‐tissue epigenetic clock sites, so the sites not found in Study 5 may be blood specific (Horvath, 2013). Of the 11, eight show an increase in DNA methylation with age and are found in CpG islands, while the three that show a decrease in DNA methylation are found in island shores. This pattern mimics the general pattern where islands gain DNA methylation and nonislands lose DNA methylation with age. Despite obvious limitations, including the relatively low density of sites assessed and the low number of overlapping sites, these 11 sites are likely candidates for important biological associations with aging in blood. However, further work is required to identify replicable epigenetic clock sites.

**Table 1:** Eleven CpG sites associated with age in blood in at least four of the eight studies examined

	Study								Distance to closest TSS	Closest TSS gene name	Chr	Relationship to island
	1	2	3	4	5	6	7	8				
cg21801378	↑	↑	■		↑		↑	■	918	CELF6	15	Island
cg22736354	↑			↑	↑	↑	↑	■	132	NHLRC1	6	Island
cg00059225	↑	↑				↑	↑	■	40	GLRA1	5	Island
cg01820374	↓	↓	■		↓		↓		403	LAG3	12	N_Shore
cg06291867	↑	↑	■			↑	↑		509	HTR7	10	Island
cg06493994	↑		■	↑	↑		↑		174	SCGN	6	Island
cg09809672		↓	■	↓	↓		↓		3	EDARADD	1	N_Shore
cg17861230	↑	↑					↑	■	309	PDE4C	19	Island
cg19722847	↓			↓	↓		↓		−185	IPO8	12	S_Shore
cg21296230		↑	■	↑			↑		332	GREM1	15	Island
cg27320127	↑	↑					↑	■	−926	KCNK12	2	Island

1: (Bell *et al*., 2012) 2: (Bocklandt *et al*., 2011) 3: (Florath *et al*., 2014) 4: (Hannum *et al*., 2013) 5: (Horvath, 2013) 6: (Numata *et al*., 2012) 7: (Weidner *et al*., 2014) 8: (Rakyan *et al*., 2011)

Sites can be positively (↑) or negatively (↓) correlated with age. Studies 3 and 8 did not indicate the direction of change, and sites found to be associated with age in those studies are indicated by a square (■).

### Causes of aging‐associated changes in DNA methylation

One of the main questions remaining in the study of DNA methylation dynamics and age, including both epigenetic drift and the epigenetic clock, is why these changes occur. A remarkable aspect of DNA methylation is that it can be modified by external factors, and in some cases, the resulting marks are heritable through cell divisions. This balance of responsiveness to stimuli and heritability results in a unique mechanism for lasting signatures of prior exposures that accumulate over the lifetime (Cortessis *et al*., 2012; Feil & Fraga, 2012). As an example of a specific exposure that has been shown to leave a lasting signature, cigarette smoke has been linked to changes in DNA methylation at the AHRR locus both in smokers and in children of smokers (Saxonov *et al*., 2006; Monick *et al*., 2012; Joubert *et al*., 2012; Shenker *et al*., 2013; Sun *et al*., 2013 Elliott *et al*., 2014; Lee *et al*., 2015; Shah *et al*., 2014). Smoking‐associated DNA methylation changes have also been found in genes involved in inflammatory networks, important candidates in the risk of age‐related diseases such as heart disease and stroke (Breitling *et al*., 2012; Dogan *et al*., 2014). Other environmental influences, such as abuse or adversities in childhood, have also been linked to stable DNA methylation differences that persist into adulthood (Meaney, 2010; Essex *et al*., 2011; Borghol *et al*., 2012; Lam *et al*., 2012; Klengel *et al*., 2013). The accumulation of these environmental exposures, either shared or unshared across individuals, would then contribute to epigenetic change with age.

In addition to the environmental signatures, DNA methylation changes with no obvious cause or pattern have been observed and ascribed to the reduced capability of faithfully maintaining epigenetic marks over cell divisions (Fraga *et al*., 2005; Martin, 2005). Changes in the functionality of epigenetic machinery in addition to exposure of the genome to environmental factors might therefore also contribute to increasing epigenetic diversity with age (Fraga & Esteller, 2007).

While evidence suggests that both environmental and stochastic factors are associated with epigenetic changes with age, it is not clear whether they contribute differently to epigenetic drift and the epigenetic clock. The hypothesized decrease in the stability of DNA methylation with age could occur randomly, resulting in epigenetic drift, or preferentially and regularly at certain sites, which would appear as epigenetic clock sites. While environmental exposures are generally thought to be highly variable across individuals, leading to diverging epigenetic patterns or epigenetic drift, shared experiences or exposures may lead to common epigenetic changes and thus influence the epigenetic clock. For example, a longitudinal study of soldiers before and after deployment to Afghanistan showed an increase in epigenetic age associated with trauma experienced in combat (Boks *et al*., 2014). However, the consistency of epigenetic clock sites across a wide variety of people implies a third effect that is suggestive of a functional or structural reason why specific sites are more likely to undergo change with age. Further studies are required to disentangle these potential contributors to epigenetic aging.

## What can we learn from DNA methylation and aging?

**Figure 2:**
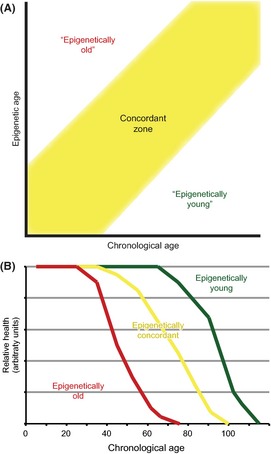
The concordance or discordance between epigenetic age and chronological age may be an indicator of future health. (A) Across a population, people can be categorized as epigenetically old, where their epigenetic age is higher than their chronological age, epigenetically young where the reverse is true, or concordant, where neither is true. (B) It is interesting to speculate whether these groups would show divergent trajectories of health as they age.

Both epigenetic drift and the epigenetic clock describe changes in DNA methylation with age, and both are almost certainly associated with age‐related phenotypes. Epigenetic drift reflects the global decrease in stability and precision of DNA methylation with age. However, as it is a (epi)genome‐wide phenomenon without consistent sites, its usefulness in terms of determining health outcomes may be limited, as longitudinal samples from the same individual would be required to track its progress. The epigenetic clock, on the other hand, has potential as a biomarker of aging as it may represent functional age‐related epigenetic changes that are common across individuals. In fact, the epigenetic clock has already been used to predict the epigenetic age of tissue samples (Horvath, 2013). In any application of epigenetic age as a biomarker, the predicted epigenetic age when compared to chronological age across many individuals would generally show a linear relationship (Fig. 2A). However, some individuals would likely be in the ‘off‐diagonal’ region of this comparison, with an epigenetic age that is higher (‘epigenetically old’) or lower (‘epigenetically young’) than their chronological age. This trend has already been observed using the age predictors described in many of the studies described in Table 1, one study in particular found that centenarians had an unusually young epigenetic age (Gentilini *et al*., 2013). An intriguing possibility is to predict health outcomes for those in the epigenetically old or epigenetically young groups relative to those who display concordant epigenetic and chronological ages, supported by a very recent study which associated accelerated epigenetic age with increased mortality (Marioni *et al*., 2015) (Fig. 2B). In this way, epigenetic age could be a more powerful predictor than chronological age for future health decline.

In addition to potential age‐related biomarkers that could predict future health, an epigenetic signature of age based on the epigenetic clock could give insight into why some age‐related phenotypes occur, thus opening areas for potential intervention and attenuation of the harmful physical manifestations of age. In a prospective study of healthy individuals, sites that gained DNA methylation with age were also found to have greater variability across women who developed cervical cancer within the following three years compared to those women who remained healthy (Teschendorff *et al*., 2012). Specific factors like smoking and obesity have been shown to increase epigenetic age in both men and women, factors which have also been shown to decrease actual life expectancy in populations (Elliott *et al*., 2014; Weidner *et al*., 2014). Recently, high body mass index was associated with an accelerated epigenetic age in liver tissue, that is, the predicted age of the liver tissue was significantly older than expected based on the donor's age; of note, this study did not replicate the finding of increased epigenetic age with smoking (Horvath *et al*., 2014). Thus, studies assessing the epigenetic clock are potentially very useful to determine which regions of the genome are targets for these changes and how this can inform interventions or lifestyle changes. However, there are a number of potential confounders and limitations, including reproducibility, for studies of epigenetics and aging that must be considered.

## Considerations for studies of epigenetics and aging

The enormous interest in epigenetics as a reporter and potentially a mediator of aging is a sign for the perpetuation of studies on DNA methylation and age. Given the current status of the field, there are a number of considerations for studies looking to assess this relationship. The first are study design and technology. While both cross‐sectional and longitudinal have advantages, the study design must be able to address the specific questions being asked about the DNA methylation and age. As epigenetic drift is distinct in each individual, longitudinal studies are necessary for explorations of epigenetic drift over age. These studies have the benefit of being able to control for differences between individuals; however, the costs and time investment can be prohibitive. In contrast, either longitudinal or cross‐sectional studies are appropriate for studies assessing the epigenetic clock. In terms of technology, the Illumina 450K array is the current gold standard for DNA methylation arrays, but it has limitations in terms of genomic coverage at regions that may potentially be important to aging, such as of repetitive elements and long noncoding RNAs. As the study of DNA methylation and aging develops, improvements in technology will begin to give us a better understanding of the genomic locations where these changes occur. For studies of epigenetic drift or the epigenetic clock, the integration of environmental exposures and health outcomes will be a particular challenge, though highly pertinent as exposures such as sun exposure and stress have already been shown to affect DNA methylation levels at age‐associated sites (Grönniger *et al*., 2010; Tapp *et al*., 2013; Boks *et al*., 2014 Noreen *et al*., 2014).

Cell composition is a major confounding factor for epigenetic studies, as cell lineage is a major determinant of DNA methylation. A recent study showed that patterns of DNA methylation associated with age can easily be confounded with signatures of specific white blood cell types, as the relative frequencies of white blood cells change over the lifespan (Weng *et al*., 2009; Jaffe & Irizarry, 2014). For example, if the frequency of T cells declines with age, then a site that is methylated in T cells may show declining methylation when comparing across ages, but this is due to the change in cell frequency rather than at the site itself. The authors of this study proposed that to be certain that specific sites are reliable, it is vital to correct for cell types across individuals, although some epigenetic clock sites have been shown to not associate with cellular composition (Talens *et al*., 2012; Horvath, 2013; Jaffe & Irizarry, 2014). While this example refers specifically to blood, the same consideration is important for analysis of any tissue sample containing more than one cell type (Guintivano *et al*., 2013; Montaño *et al*., 2013). In the future, studies of age and DNA methylation will need to either include cell counts, use reference‐free cell admixture control, or, at the very least, check their age‐related hits against regions known to differ across cell types in order for results to be reliable. It should be noted, however, that the correction methods will not identify age‐related signatures that are present in a cell type that constitutes a small fraction of the tissue being studied, as these will still be overwhelmed by signals from more frequent cell types. In that case, simply separating the tissue of interest into its component cells and analyzing them individually will be necessary to identify such patterns. It is important to note that many of the studies discussed in this review have not been adjusted for differences in cellular composition with age. Therefore, tissue‐specific epigenetic clock sites in some of these studies may not be accurate; however, the overall patterns of DNA methylation change with age have been validated in studies that did correct for cellular composition (Lam *et al*., 2012; Lister *et al*., 2013; Jaffe & Irizarry, 2014).

Another consideration is the effect of genetic variation on the relationship between epigenetics and age, about which very little is currently known. Genetic variation has been shown to contribute to longevity, evidenced by the fact that longevity runs in families (Schoenmaker *et al*., 2006). Clear relationships also exist between DNA methylation and genetic variability, and recent studies have identified genetic loci whose variation is strongly associated with DNA methylation at nearby sites (Fraser *et al*., 2012; Gamazon *et al*., 2013; Gutierrez Arcelus *et al*., 2013; Moen *et al*., 2013). Thus, it is possible that genetic variants associated with longevity may also be correlated with DNA methylation changes. At this point, however, it is not clear whether these would be limited to site‐specific DNA methylation changes or whether genetic variants may affect the function of DNA methylation machinery, resulting in differences in rates of epigenetic drift.

Further evidence for genetic contributions to epigenetic aging come from studies examining age and DNA methylation that have also observed high correlation of DNA methylation levels between twins at more than one age (Bell *et al*., 2012; Martino *et al*., 2013). Similar studies in adults have shown greater concordance in DNA methylation profiles between monozygotic twins than dizygotic twins (Kaminsky *et al*., 2009; Bell *et al*., 2011). One study examined twins to determine the heritability of discordance between epigenetic and chronological age and determined that at birth, the heritability is 100% but drops to 39% in adulthood (Horvath, 2013). These findings support the idea that genetic heritability of DNA methylation is overcome by environmental perturbations during the lifespan. Hence, genetic variants that affect DNA methylation levels can do so throughout the aging process, but the effects may vary at different times during the lifespan.

Finally, it is important to consider the causative versus correlative role of DNA methylation with respect to its relationship with age. As yet, it is unknown what function, if any, DNA methylation changes with age have. An appealing hypothesis is that epigenetic drift is a marker of age, suggesting that increases in variability with age are a by‐product of the aging process itself. The consistency of the epigenetic clock, however, points to a common age‐related epigenetic mechanism across individuals. It is thus possible that these common changes are important contributors to the aging process, rather than a consequence, or they could possibly be beneficial adaptive changes occurring as a response to aging. Future work addressing this hypothesis is necessary before we can fully understand the role of DNA methylation changes that occur with age.

## Concluding remarks

DNA methylation changes that are associated with age can be considered part of two related phenomena, epigenetic drift and the epigenetic clock. We have defined epigenetic drift as the global tendency toward median DNA methylation caused by stochastic and environmental individual‐specific changes over the lifetime. The epigenetic clock, on the other hand, refers to specific sites in the genome that have been shown to undergo age‐related change across individuals and, in some cases, across tissues. We hope that this clarification of terminology will improve our understanding of age‐related DNA methylation changes as well as clarify the labeling of these distinct phenomena across researchers in the field.

A number of aspects of age‐related DNA methylation remain, which should be further scrutinized. First, it is expected that certain life periods, such as early childhood, puberty, and advanced age, result in accelerated epigenetic changes. Most studies of DNA methylation and age have examined changes within specific periods of life—the first few years of life or adulthood to old age, for example. Moving forward, it will be important to determine what periods during the lifespan are the most changeable, which highlights the need for more rigorous studies. Moreover, work on the effects of environmental stimuli on the rates of epigenetic aging would contribute insight into how or why specific environmental exposures result in increased mortality. It could be hypothesized that people who are exposed to factors that affect mortality show advanced epigenetic compared to chronological age, although these effects may be tissue specific. Additionally, several recent cross‐sectional studies have published epigenetic clocks. Comparison of these sites across longitudinal studies, while controlling for confounders in DNA methylation such as tissue type, cellular composition, ethnicity, and environment, is necessary to confirm a consistent, reliable, and independent signature of DNA methylation and aging. This type of age predictor could be of use in a number of areas. One potential application is in forensics, where the ability to determine the approximate age of a person from a biological sample would be invaluable (Lee *et al*., 2012). In health, epigenetic age could be used to target or assess interventions or treatments. However, the health‐related potential of epigenetic age still waits on an assessment of concordance between epigenetic and chronological age across a large population with longitudinal tracking of health during the aging process. This field has immense potential to inform human populations and will undoubtedly continue to develop in the near future.

## Funding

No funding information provided.

## Conflict of interest

The authors declare that they have no conflict of interest.

## References

[acel12349-bib-0001] Adkins RM , Thomas F , Tylavsky FA , Krushkal J (2011) Parental ages and levels of DNA methylation in the newborn are correlated. BMC Med. Genet. 12, 47. 21453505 10.1186/1471-2350-12-47 PMC3078852

[acel12349-bib-0002] Alisch RS , Barwick BG , Chopra P , Myrick LK , Satten GA , Conneely KN , Warren ST (2012) Age‐associated DNA methylation in pediatric populations. Genome Res. 22, 623–632. 22300631 10.1101/gr.125187.111 PMC3317145

[acel12349-bib-0003] Alves G , Tatro A , Fanning T (1996) Differential methylation of human LINE‐1 retrotransposons in malignant cells. Gene 176, 39–44. 8918229 10.1016/0378-1119(96)00205-3

[acel12349-bib-0004] Baylin SB , Jones PA (2011) A decade of exploring the cancer epigenome ‐ biological and translational implications. Nat. Rev. Cancer 11, 726–734. 21941284 10.1038/nrc3130 PMC3307543

[acel12349-bib-0005] Bell JT , Pai AA , Pickrell JK , Gaffney DJ , Pique‐Regi R , Degner JF , Gilad Y , Pritchard JK (2011) DNA methylation patterns associate with genetic and gene expression variation in HapMap cell lines. Genome Biol. 12, R10. 21251332 10.1186/gb-2011-12-1-r10 PMC3091299

[acel12349-bib-0006] Bell JT , Tsai P‐C , Yang T‐P , Pidsley R , Nisbet J , Glass D , Mangino M , Zhai G , Zhang F , Valdes A , Shin S‐Y , Dempster EL , Murray RM , Grundberg E , Hedman AK , Nica A , Small KS , Dermitzakis ET , McCarthy MI , Mill J , Spector TD , Deloukas P , Consortium M (2012) Epigenome‐wide scans identify differentially methylated regions for age and age‐related phenotypes in a healthy ageing population. PLoS Genet. 8, 189–200. 10.1371/journal.pgen.1002629 PMC3330116 22532803

[acel12349-bib-0007] Bergman Y , Cedar H (2013) DNA methylation dynamics in health and disease. Nat. Struct. Mol. Biol. 20, 274–281. 23463312 10.1038/nsmb.2518

[acel12349-bib-0008] Bird A (2007) Perceptions of epigenetics. Nature 447, 396–398. 17522671 10.1038/nature05913

[acel12349-bib-0009] Bjornsson HT , Fallin MD , Feinberg AP (2004) An integrated epigenetic and genetic approach to common human disease. Trends Genet. 20, 350–358. 15262407 10.1016/j.tig.2004.06.009

[acel12349-bib-0010] Bocklandt S , Lin W , Sehl ME , Sanchez FJ , Sinsheimer JS , Horvath S , Vilain E (2011) Epigenetic predictor of age. PLoS ONE 6, e14821. 21731603 10.1371/journal.pone.0014821 PMC3120753

[acel12349-bib-0011] Boks MP , Derks EM , Weisenberger DJ , Strengman E , Janson E , Sommer IE , Kahn RS , Ophoff RA (2009) The relationship of DNA methylation with age, gender and genotype in twins and healthy controls. PLoS ONE 4, e6767. 19774229 10.1371/journal.pone.0006767 PMC2747671

[acel12349-bib-0012] Boks MP , Mierlo HCV , Rutten BPF , Radstake TRDJ , De Witte L , Geuze E , Horvath S , Schalkwyk LC , Vinkers CH , Broen JCA , Vermetten E (2014) Longitudinal changes of telomere length and epigenetic age related to traumatic stress and post‐traumatic stress disorder. Psychoneuroendocrinology 51, 506–512. 25129579 10.1016/j.psyneuen.2014.07.011

[acel12349-bib-0013] Bollati V , Schwartz J , Wright R , Litonjua A , Tarantini L , Suh H , Sparrow D , Vokonas P , Baccarelli A (2009) Decline in genomic DNA methylation through aging in a cohort of elderly subjects. Mech. Ageing Dev. 130, 234–239. 19150625 10.1016/j.mad.2008.12.003 PMC2956267

[acel12349-bib-0014] Borghol N , Suderman M , McArdle W , Racine A , Hallett M , Pembrey M , Hertzman C , Power C , Szyf M (2012) Associations with early‐life socio‐economic position in adult DNA methylation. Int. J. Epidemiol. 41, 62–74. 22422449 10.1093/ije/dyr147 PMC3304522

[acel12349-bib-0015] Breitling LP , Salzmann K , Rothenbacher D , Burwinkel B , Brenner H (2012) Smoking, F2RL3 methylation, and prognosis in stable coronary heart disease. Eur. Heart J. 33, 2841–2848. 22511653 10.1093/eurheartj/ehs091

[acel12349-bib-0016] Byun H‐M , Siegmund KD , Pan F , Weisenberger DJ , Kanel G , Laird PW , Yang AS (2009) Epigenetic profiling of somatic tissues from human autopsy specimens identifies tissue‐ and individual‐specific DNA methylation patterns. Hum. Mol. Genet. 18, 4808–4817. 19776032 10.1093/hmg/ddp445 PMC4481584

[acel12349-bib-0017] Cooney CA (1993) Are somatic‐cells inherently deficient in methylation metabolism ‐ a proposed mechanism for DNA methylation loss, senescence and aging. J. Cell. Biochem. 17D, 164–164. 8300279

[acel12349-bib-0018] Cortessis VK , Thomas DC , Levine AJ , Breton CV , Mack TM , Siegmund KD , Haile RW , Laird PW (2012) Environmental epigenetics: prospects for studying epigenetic mediation of exposure‐response relationships. Hum. Genet. 131, 1565–1589. 22740325 10.1007/s00439-012-1189-8 PMC3432200

[acel12349-bib-0019] Davies MN , Volta M , Pidsley R , Lunnon K , Dixit A , Lovestone S , Coarfa C , Harris RA , Milosavljevic A , Troakes C , Al‐Sarraj S , Dobson R , Schalkwyk LC , Mill J (2012) Functional annotation of the human brain methylome identifies tissue‐specific epigenetic variation across brain and blood. Genome Biol. 13, R43. 22703893 10.1186/gb-2012-13-6-r43 PMC3446315

[acel12349-bib-0020] Day K , Waite LL , Thalacker‐Mercer A , West A , Bamman MM , Brooks JD , Myers RM , Absher D (2013) Differential DNA methylation with age displays both common and dynamic features across human tissues that are influenced by CpG landscape. Genome Biol. 14, R102. 24034465 10.1186/gb-2013-14-9-r102 PMC4053985

[acel12349-bib-0021] Dogan MV , Shields B , Cutrona C , Gao L , Gibbons FX , Simons R , Monick M , Brody GH , Tan K , Beach SRH , Philibert RA (2014) The effect of smoking on DNA methylation of peripheral blood mononuclear cells from African American women. BMC Genom. 15, 151. 10.1186/1471-2164-15-151 PMC3936875 24559495

[acel12349-bib-0022] Drinkwater RD , Blake TJ , Morley AA , Turner DR (1989) Human lymphocytes aged in vivo have reduced levels of methylation in transcriptionally active and inactive DNA. Mutat. Res. 219, 29–37. 2911269 10.1016/0921-8734(89)90038-6

[acel12349-bib-0023] Egger G , Liang G , Aparicio A , Jones PA (2004) Epigenetics in human disease and prospects for epigenetic therapy. Nature 429, 457–463. 15164071 10.1038/nature02625

[acel12349-bib-0024] van Eijk KR , de Jong S , Boks MPM , Langeveld T , Colas F , Veldink JH , de Kovel CGF , Janson E , Strengman E , Langfelder P , Kahn RS , van den Berg LH , Horvath S , Ophoff RA (2012) Genetic analysis of DNA methylation and gene expression levels in whole blood of healthy human subjects. BMC Genom. 13, 636. 10.1186/1471-2164-13-636 PMC3583143 23157493

[acel12349-bib-0025] Elliott HR , Tillin T , McArdle WL , Ho K , Duggirala A , Frayling TM , Davey Smith G , Hughes AD , Chaturvedi N , Relton CL (2014) Differences in smoking associated DNA methylation patterns in South Asians and Europeans. Clin. Epigenetics 6, 4. 24485148 10.1186/1868-7083-6-4 PMC3915234

[acel12349-bib-0026] Essex MJ , Thomas Boyce W , Hertzman C , Lam LL , Armstrong JM , Neumann SMA , Kobor MS (2011) Epigenetic vestiges of early developmental adversity: childhood stress exposure and DNA methylation in adolescence. Child Dev. 00, 1–18. 10.1111/j.1467-8624.2011.01641.x PMC3235257 21883162

[acel12349-bib-0027] Feil R , Fraga MF (2012) Epigenetics and the environment: emerging patterns and implications. Nat. Rev. Genet. 13, 97–109. 22215131 10.1038/nrg3142

[acel12349-bib-0028] Florath I , Butterbach K , Müller H , Bewerunge‐Hudler M , Brenner H (2014) Cross‐sectional and longitudinal changes in DNA methylation with age: an epigenome‐wide analysis revealing over 60 novel age‐associated CpG sites. Hum. Mol. Genet. 23, 1186–1201. 24163245 10.1093/hmg/ddt531 PMC3919014

[acel12349-bib-0029] Fraga MF , Esteller M (2007) Epigenetics and aging: the targets and the marks. Trends Genet. 23, 413–418. 17559965 10.1016/j.tig.2007.05.008

[acel12349-bib-0030] Fraga MF , Ballestar E , Paz MF , Ropero S , Setien F , Ballestart ML , Heine‐Suner D , Cigudosa JC , Urioste M , Benitez J , Boix‐Chornet M , Sanchez‐Aguilera A , Ling C , Carlsson E , Poulsen P , Vaag A , Stephan Z , Spector TD , Wu YZ , Plass C , Esteller M (2005) Epigenetic differences arise during the lifetime of monozygotic twins. Proc. Natl Acad. Sci. USA 102, 10604–10609. 16009939 10.1073/pnas.0500398102 PMC1174919

[acel12349-bib-0031] Fraser HB , Lam LL , Neumann SM , Kobor MS (2012) Population‐specificity of human DNA methylation. Genome Biol. 13, R8. 22322129 10.1186/gb-2012-13-2-r8 PMC3334571

[acel12349-bib-0032] Fuke C , Shimabukuro M , Petronis A , Sugimoto J , Oda T , Miura K , Miyazaki T , Ogura C , Okazaki Y , Jinno Y (2004) Age related changes in 5‐methylcytosine content in human peripheral leukocytes and placentas: an HPLC‐based study. Ann. Hum. Genet. 68, 196–204. 15180700 10.1046/j.1529-8817.2004.00081.x

[acel12349-bib-0033] Gamazon ER , Badner JA , Cheng L , Zhang C , Zhang D , Cox NJ , Gershon ES , Kelsoe JR , Greenwood TA , Nievergelt CM , Chen C , McKinney R , Shilling PD , Schork NJ , Smith EN , Bloss CS , Nurnberger JI , Edenberg HJ , Foroud T , Koller DL , Scheftner WA , Coryell W , Rice J , Lawson WB , Nwulia EA , Hipolito M , Byerley W , McMahon FJ , Schulze TG , Berrettini WH , Potash JB , Zandi PP , Mahon PB , McInnis MG , Zöllner S , Zhang P , Craig DW , Szelinger S , Barrett TB , Liu C (2013) Enrichment of cis‐regulatory gene expression SNPs and methylation quantitative trait loci among bipolar disorder susceptibility variants. Mol. Psychiatry 18, 340–346. 22212596 10.1038/mp.2011.174 PMC3601550

[acel12349-bib-0034] Gentilini D , Mari D , Castaldi D , Remondini D , Ogliari G , Ostan R , Bucci L , Sirchia SM , Tabano S , Cavagnini F , Monti D , Franceschi C , Di Blasio AM , Vitale G (2013) Role of epigenetics in human aging and longevity: genome‐wide DNA methylation profile in centenarians and centenarians' offspring. Age 35, 1961–1973. 22923132 10.1007/s11357-012-9463-1 PMC3776126

[acel12349-bib-0035] Grönniger E , Weber B , Heil O , Peters N , Stäb F , Wenck H , Korn B , Winnefeld M , Lyko F (2010) Aging and chronic sun exposure cause distinct epigenetic changes in human skin. PLoS Genet. 6, e1000971. 20523906 10.1371/journal.pgen.1000971 PMC2877750

[acel12349-bib-0036] Guintivano J , Aryee MJ , Kaminsky ZA (2013) A cell epigenotype specific model for the correction of brain cellular heterogeneity bias and its application to age, brain region and major depression. Epigenetics 8, 290–302. 23426267 10.4161/epi.23924 PMC3669121

[acel12349-bib-0037] Gutierrez Arcelus M , Lappalainen T , Montgomery SB , Buil A , Ongen H , Yurovsky A , Bryois J , Giger T , Romano L , Planchon A , Falconnet E , Bielser D , Gagnebin M , Padioleau I , Borel C , Letourneau A , Makrythanasis P , Guipponi M , Gehrig C , Antonarakis SE , Dermitzakis ET (2013) Passive and active DNA methylation and the interplay with genetic variation in gene regulation. Elife 2, e00523. 23755361 10.7554/eLife.00523 PMC3673336

[acel12349-bib-0038] Hannum G , Guinney J , Zhao L , Zhang L , Hughes G , Sadda S , Klotzle B , Bibikova M , Fan J‐B , Gao Y , Deconde R , Chen M , Rajapakse I , Friend S , Ideker T , Zhang K (2013) Genome‐wide Methylation Profiles Reveal Quantitative Views of Human Aging Rates. Mol. Cell 49, 359–367. 23177740 10.1016/j.molcel.2012.10.016 PMC3780611

[acel12349-bib-0039] Herbstman JB , Wang S , Perera FP , Lederman SA , Vishnevetsky J , Rundle AG , Hoepner LA , Qu L , Tang D (2013) Predictors and consequences of global DNA methylation in cord blood and at three years. PLoS ONE 8, e72824. 24023780 10.1371/journal.pone.0072824 PMC3762861

[acel12349-bib-0040] Heyn H , Li N , Ferreira HJ , Moran S , Pisano DG , Gomez A , Diez J , Sanchez‐Mut JV , Setien F , Javier Carmona F , Puca AA , Sayols S , Pujana MA , Serra‐Musach J , Iglesias‐Platas I , Formiga F , Fernandez AF , Fraga MF , Heath SC , Valencia A , Gut IG , Wang J , Esteller M (2012) Distinct DNA methylomes of newborns and centenarians. Proc. Natl Acad. Sci. USA 109, 10522–10527. 22689993 10.1073/pnas.1120658109 PMC3387108

[acel12349-bib-0041] Horvath S (2013) DNA methylation age of human tissues and cell types. Genome Biol. 14, R115. 24138928 10.1186/gb-2013-14-10-r115 PMC4015143

[acel12349-bib-0042] Horvath S , Zhang Y , Langfelder P , Kahn RS , Boks MP , van Eijk K , van den Berg LH , Ophoff RA (2012) Aging effects on DNA methylation modules in human brain and blood tissue. Genome Biol. 13, R97. 23034122 10.1186/gb-2012-13-10-r97 PMC4053733

[acel12349-bib-0043] Horvath S , Erhart W , Brosch M , Ammerpohl O , von Schönfels W , Ahrens M , Heits N , Bell JT , Tsai P‐C , Spector TD , Deloukas P , Siebert R , Sipos B , Becker T , Röcken C , Schafmayer C , Hampe J (2014) Obesity accelerates epigenetic aging of human liver. Proc. Natl. Acad. Sci. USA 111, 15538–15543. 25313081 10.1073/pnas.1412759111 PMC4217403

[acel12349-bib-0044] Humpherys D , Eggan K , Akutsu H , Hochedlinger K , Rideout WM , Biniszkiewicz D , Yanagimachi R , Jaenisch R (2001) Epigenetic instability in ES cells and cloned mice. Science 293, 95–97. 11441181 10.1126/science.1061402

[acel12349-bib-0045] Illingworth RS , Bird AP (2009) CpG islands–'a rough guide'. FEBS Lett. 583, 1713–1720. 19376112 10.1016/j.febslet.2009.04.012

[acel12349-bib-0046] Jaffe AE , Irizarry RA (2014) Accounting for cellular heterogeneity is critical in epigenome‐wide association studies. Genome Biol. 15, R31. 24495553 10.1186/gb-2014-15-2-r31 PMC4053810

[acel12349-bib-0047] Jenkins TG , Aston KI , Cairns BR , Carrell DT (2013) Paternal aging and associated intraindividual alterations of global sperm 5‐methylcytosine and 5‐hydroxymethylcytosine levels. Fertil. Steril. 100, 945–951. 23809503 10.1016/j.fertnstert.2013.05.039

[acel12349-bib-0048] Jiang R , Jones MJ , Chen E , Neumann SM , Fraser HB , Miller GE , Kobor MS (2015) Discordance of DNA methylation variance between two accessible human tissues. Sci. Rep. 5, 8257. 25660083 10.1038/srep08257 PMC4321176

[acel12349-bib-0049] Johansson A , Enroth S , Gyllensten U (2013) Continuous Aging of the Human DNA Methylome Throughout the Human Lifespan. PLoS ONE 8, e67378. 23826282 10.1371/journal.pone.0067378 PMC3695075

[acel12349-bib-0050] Jones PA (2012) Functions of DNA methylation: islands, start sites, gene bodies and beyond. Nat. Rev. Genet. 13, 484–492. 22641018 10.1038/nrg3230

[acel12349-bib-0051] Joubert BR , Håberg SE , Nilsen RM , Wang X , Vollset SE , Murphy SK , Huang Z , Hoyo C , Midttun Ø , Cupul‐Uicab LA , Ueland PM , Wu MC , Nystad W , Bell DA , Peddada SD , London SJ (2012) 450K epigenome‐wide scan identifies differential DNA methylation in newborns related to maternal smoking during pregnancy. Environ. Health Perspect. 120, 1425–1431. 22851337 10.1289/ehp.1205412 PMC3491949

[acel12349-bib-0052] Kaminsky ZA , Tang T , Wang S‐C , Ptak C , Oh GHT , Wong AHC , Feldcamp LA , Virtanen C , Halfvarson J , Tysk C , McRae AF , Visscher PM , Montgomery GW , Gottesman II , Martin NG , Petronis A (2009) DNA methylation profiles in monozygotic and dizygotic twins. Nat. Genet. 41, 240–245. 19151718 10.1038/ng.286

[acel12349-bib-0053] Klengel T , Mehta D , Anacker C , Rex‐Haffner M , Pruessner JC , Pariante CM , Pace TWW , Mercer KB , Mayberg HS , Bradley B , Nemeroff CB , Holsboer F , Heim CM , Ressler KJ , Rein T , Binder EB (2013) Allele‐specific FKBP5 DNA demethylation mediates gene‐childhood trauma interactions. Nat. Neurosci. 16, 33–41. 23201972 10.1038/nn.3275 PMC4136922

[acel12349-bib-0054] Kochanek S , Renz D , Doerfler W (1993) DNA methylation in the Alu sequences of diploid and haploid primary human cells. EMBO J. 12, 1141–1151. 8384552 10.1002/j.1460-2075.1993.tb05755.x PMC413315

[acel12349-bib-0055] Kwabi‐Addo B , Chung W , Shen L , Ittmann M , Wheeler T , Jelinek J , Issa J‐PJ (2007) Age‐related DNA methylation changes in normal human prostate tissues. Clin. Cancer Res. 13, 3796–3802. 17606710 10.1158/1078-0432.CCR-07-0085

[acel12349-bib-0056] Lam LL , Emberly E , Fraser HB , Neumann SM , Chen E , Miller GE , Kobor MS (2012) Factors underlying variable DNA methylation in a human community cohort. Proc. Natl. Acad. Sci. USA 109(Suppl 2), 17253–17260. 23045638 10.1073/pnas.1121249109 PMC3477380

[acel12349-bib-0057] Lee HY , Park MJ , Choi A , An JH , Yang WI , Shin K‐J (2012) Potential forensic application of DNA methylation profiling to body fluid identification. Int. J. Legal Med. 126, 55–62. 21626087 10.1007/s00414-011-0569-2

[acel12349-bib-0058] Lee KWK , Richmond R , Hu P , French L , Shin J , Bourdon C , Reischl E , Waldenberger M , Zeilinger S , Gaunt T , McArdle W , Ring S , Woodward G , Bouchard L , Gaudet D , Davey Smith G , Relton C , Paus T , Pausova Z (2015) Prenatal exposure to maternal cigarette smoking and DNA methylation: epigenome‐wide association in a discovery sample of adolescents and replication in an independent cohort at birth through 17 years of age. Environ. Health Perspect. 123, 193–199. 25325234 10.1289/ehp.1408614 PMC4314251

[acel12349-bib-0059] Lister R , Pelizzola M , Dowen RH , Hawkins RD , Hon G , Tonti‐Filippini J , Nery JR , Lee L , Ye Z , Ngo Q‐M , Edsall L , Antosiewicz‐Bourget J , Stewart R , Ruotti V , Millar AH , Thomson JA , Ren B , Ecker JR (2009) Human DNA methylomes at base resolution show widespread epigenomic differences. Nature 462, 315–322. 19829295 10.1038/nature08514 PMC2857523

[acel12349-bib-0060] Lister R , Mukamel EA , Nery JR , Urich M , Puddifoot CA , Johnson ND , Lucero J , Huang Y , Dwork AJ , Schultz MD , Yu M , Tonti‐Filippini J , Heyn H , Hu S , Wu JC , Rao A , Esteller M , He C , Haghighi FG , Sejnowski TJ , Behrens MM , Ecker JR (2013) Global epigenomic reconfiguration during mammalian brain development. Science 341, 1237905. 23828890 10.1126/science.1237905 PMC3785061

[acel12349-bib-0061] Lopez‐Otin C , Blasco MA , Partridge L , Serrano M , Kroemer G (2013) The Hallmarks of Aging. Cell 153, 1194–1217. 23746838 10.1016/j.cell.2013.05.039 PMC3836174

[acel12349-bib-0062] Marioni RE , Shah S , McRae AF , Chen BH , Colicino E , Harris SE , Gibson J , Henders AK , Redmond P , Cox SR , Pattie A , Corley J , Murphy L , Martin NG , Montgomery GW , Feinberg AP , Fallin M , Multhaup ML , Jaffe AE , Joehanes R , Schwartz J , Just AC , Lunetta KL , Murabito JM , Starr JM , Horvath S , Baccarelli AA , Levy D , Visscher PM , Wray NR , Deary IJ (2015) DNA methylation age of blood predicts all‐cause mortality in later life. Genome Biol. 16, 25. 25633388 10.1186/s13059-015-0584-6 PMC4350614

[acel12349-bib-0063] Martin GM (2005) Epigenetic drift in aging identical twins. Proc. Natl. Acad. Sci. USA 102, 10413–10414. 16027353 10.1073/pnas.0504743102 PMC1180801

[acel12349-bib-0064] Martino DJ , Tulic MK , Gordon L , Hodder M , Richman TR , Metcalfe J , Prescott SL , Saffery R (2011) Evidence for age‐related and individual‐specific changes in DNA methylation profile of mononuclear cells during early immune development in humans. Epigenetics 6, 1085–1094. 21814035 10.4161/epi.6.9.16401

[acel12349-bib-0065] Martino D , Loke YJ , Gordon L , Ollikainen M , Cruickshank MN , Saffery R , Craig JM (2013) Longitudinal, genome‐scale analysis of DNA methylation in twins from birth to 18 months of age reveals rapid epigenetic change in early life and pair‐specific effects of discordance. Genome Biol. 14, R42. 23697701 10.1186/gb-2013-14-5-r42 PMC4054827

[acel12349-bib-0066] McClay JL , Aberg KA , Clark SL , Nerella S , Kumar G , Xie LY , Hudson AD , Harada A , Hultman CM , Magnusson PKE , Sullivan PF , van den Oord EJCG (2014) A methylome‐wide study of aging using massively parallel sequencing of the methyl‐CpG‐enriched genomic fraction from blood in over 700 subjects. Hum. Mol. Genet. 23, 1175–1185. 24135035 10.1093/hmg/ddt511 PMC3919012

[acel12349-bib-0067] Meaney MJ (2010) Epigenetics and the biological definition of gene x environment interactions. Child Dev. 81, 41–79. 20331654 10.1111/j.1467-8624.2009.01381.x

[acel12349-bib-0068] Moen EL , Zhang X , Mu W , Delaney SM , Wing C , McQuade J , Myers J , Godley LA , Dolan ME , Zhang W (2013) Genome‐wide variation of cytosine modifications between European and African populations and the implications for complex traits. Genetics 194, 987–996. 23792949 10.1534/genetics.113.151381 PMC3730924

[acel12349-bib-0069] Monick MM , Beach SRH , Plume J , Sears R , Gerrard M , Brody GH , Philibert RA (2012) Coordinated changes in AHRR methylation in lymphoblasts and pulmonary macrophages from smokers. Am. J. Med. Genet. B Neuropsychiatr. Genet. 159B, 141–151. 22232023 10.1002/ajmg.b.32021 PMC3318996

[acel12349-bib-0070] Montaño CM , Irizarry RA , Kaufmann WE , Talbot K , Gur RE , Feinberg AP , Taub MA (2013) Measuring cell‐type specific differential methylation in human brain tissue. Genome Biol. 14, R94. 24000956 10.1186/gb-2013-14-8-r94 PMC4054676

[acel12349-bib-0071] Noreen F , Röösli M , Gaj P , Pietrzak J , Weis S , Urfer P , Regula J , Schär P , Truninger K (2014) Modulation of age‐ and cancer‐associated DNA methylation change in the healthy colon by aspirin and lifestyle. J. Natl Cancer Inst. 106. 10.1093/jnci/dju161 PMC4112799 24973978

[acel12349-bib-0072] Numata S , Ye T , Hyde TM , Guitart‐Navarro X , Tao R , Wininger M , Colantuoni C , Weinberger DR , Kleinman JE , Lipska BK (2012) DNA methylation signatures in development and aging of the human prefrontal cortex. Am. J. Hum. Genet. 90, 260–272. 22305529 10.1016/j.ajhg.2011.12.020 PMC3276664

[acel12349-bib-0073] Ong M‐L , Holbrook JD (2014) Novel region discovery method for Infinium 450K DNA methylation data reveals changes associated with aging in muscle and neuronal pathways. Aging Cell 13, 142–155. 24112369 10.1111/acel.12159 PMC4326857

[acel12349-bib-0074] Poulsen P , Esteller M , Vaag A , Fraga MF (2007) The epigenetic basis of twin discordance in age‐related diseases. Pediatr. Res. 61, 38R–42R. 10.1203/pdr.0b013e31803c7b98 17413848

[acel12349-bib-0075] Rakyan VK , Down TA , Maslau S , Andrew T , Yang T‐P , Beyan H , Whittaker P , McCann OT , Finer S , Valdes AM , Leslie RD , Deloukas P , Spector TD (2010) Human aging‐associated DNA hypermethylation occurs preferentially at bivalent chromatin domains. Genome Res. 20, 434–439. 20219945 10.1101/gr.103101.109 PMC2847746

[acel12349-bib-0076] Rakyan VK , Down TA , Balding DJ , Beck S (2011) Epigenome‐wide association studies for common human diseases. Nat. Rev. Genet. 12, 529–541. 21747404 10.1038/nrg3000 PMC3508712

[acel12349-bib-0077] Saxonov S , Berg P , Brutlag DL (2006) A genome‐wide analysis of CpG dinucleotides in the human genome distinguishes two distinct classes of promoters. Proc. Natl. Acad. Sci. USA 103, 1412–1417. 16432200 10.1073/pnas.0510310103 PMC1345710

[acel12349-bib-0078] Schoenmaker M , de Craen AJM , de Meijer PHEM , Beekman M , Blauw GJ , Slagboom PE , Westendorp RGJ (2006) Evidence of genetic enrichment for exceptional survival using a family approach: the Leiden Longevity Study. Eur. J. Hum. Genet. 14, 79–84. 16251894 10.1038/sj.ejhg.5201508

[acel12349-bib-0079] Shah S , McRae AF , Marioni RE , Harris SE , Gibson J , Henders AK , Redmond P , Cox SR , Pattie A , Corley J , Murphy L , Martin NG , Montgomery GW , Starr JM , Wray NR , Deary IJ , Visscher PM (2014) Genetic and environmental exposures constrain epigenetic drift over the human life course. Genome Res. 24, 1725–1733. 25249537 10.1101/gr.176933.114 PMC4216914

[acel12349-bib-0080] Shenker NS , Polidoro S , van Veldhoven K , Sacerdote C , Ricceri F , Birrell MA , Belvisi MG , Brown R , Vineis P , Flanagan JM (2013) Epigenome‐wide association study in the European Prospective Investigation into Cancer and Nutrition (EPIC‐Turin) identifies novel genetic loci associated with smoking. Hum. Mol. Genet. 22, 843–851. 23175441 10.1093/hmg/dds488

[acel12349-bib-0081] Sun YV , Smith AK , Conneely KN , Chang Q , Li W , Lazarus A , Smith JA , Almli LM , Binder EB , Klengel T , Cross D , Turner ST , Ressler KJ , Kardia SLR (2013) Epigenomic association analysis identifies smoking‐related DNA methylation sites in African Americans. Hum. Genet. 132, 1027–1037. 23657504 10.1007/s00439-013-1311-6 PMC3744600

[acel12349-bib-0082] Talens RP , Christensen K , Putter H , Willemsen G , Christiansen L , Kremer D , Suchiman HED , Slagboom PE , Boomsma DI , Heijmans BT (2012) Epigenetic variation during the adult lifespan: cross‐sectional and longitudinal data on monozygotic twin pairs. Aging Cell 11, 694–703. 22621408 10.1111/j.1474-9726.2012.00835.x PMC3399918

[acel12349-bib-0083] Tapp HS , Commane DM , Bradburn DM , Arasaradnam R , Mathers JC , Johnson IT , Belshaw NJ (2013) Nutritional factors and gender influence age‐related DNA methylation in the human rectal mucosa. Aging Cell 12, 148–155. 23157586 10.1111/acel.12030 PMC3572581

[acel12349-bib-0084] Teschendorff AE , Menon U , Gentry‐Maharaj A , Ramus SJ , Weisenberger DJ , Shen H , Campan M , Noushmehr H , Bell CG , Maxwell AP , Savage DA , Mueller‐Holzner E , Marth C , Kocjan G , Gayther SA , Jones A , Beck S , Wagner W , Laird PW , Jacobs IJ , Widschwendter M (2010) Age‐dependent DNA methylation of genes that are suppressed in stem cells is a hallmark of cancer. Genome Res. 20, 440–446. 20219944 10.1101/gr.103606.109 PMC2847747

[acel12349-bib-0085] Teschendorff AE , Jones A , Fiegl H , Sargent A , Zhuang JJ , Kitchener HC , Widschwendter M (2012) Epigenetic variability in cells of normal cytology is associated with the risk of future morphological transformation. Genome Med. 4, 24. 22453031 10.1186/gm323 PMC3446274

[acel12349-bib-0086] Teschendorff AE , West J , Beck S (2013) Age‐associated epigenetic drift: implications, and a case of epigenetic thrift? Hum. Mol. Genet. 22, 7–15. 10.1093/hmg/ddt375 PMC3782071 23918660

[acel12349-bib-0087] Viré E , Brenner C , Deplus R , Blanchon L , Fraga M , Didelot C , Morey L , Van Eynde A , Bernard D , Vanderwinden J‐M , Bollen M , Esteller M , Di Croce L , de Launoit Y , Fuks F (2006) The Polycomb group protein EZH2 directly controls DNA methylation. Nature 439, 871–874. 16357870 10.1038/nature04431

[acel12349-bib-0088] Wagner JR , Busche S , Ge B , Kwan T , Pastinen T , Blanchette M (2014) The relationship between DNA methylation, genetic and expression inter‐individual variation in untransformed human fibroblasts. Genome Biol. 15, R37. 24555846 10.1186/gb-2014-15-2-r37 PMC4053980

[acel12349-bib-0089] Wang T , Pan Q , Lin L , Szulwach KE , Song C‐X , He C , Wu H , Warren ST , Jin P , Duan R , Li X (2012) Genome‐wide DNA hydroxymethylation changes are associated with neurodevelopmental genes in the developing human cerebellum. Hum. Mol. Genet. 21, 5500–5510. 23042784 10.1093/hmg/dds394 PMC3516134

[acel12349-bib-0090] Weber M , Hellmann I , Stadler MB , Ramos L , Pääbo S , Rebhan M , Schübeler D (2007) Distribution, silencing potential and evolutionary impact of promoter DNA methylation in the human genome. Nat. Genet. 39, 457–466. 17334365 10.1038/ng1990

[acel12349-bib-0091] Weidner CI , Lin Q , Koch CM , Eisele L , Beier F , Ziegler P , Bauerschlag DO , Jöckel K‐H , Erbel R , Mühleisen TW , Zenke M , Brümmendorf TH , Wagner W (2014) Aging of blood can be tracked by DNA methylation changes at just three CpG sites. Genome Biol. 15, R24. 24490752 10.1186/gb-2014-15-2-r24 PMC4053864

[acel12349-bib-0092] Weng N‐P , Akbar AN , Goronzy J (2009) CD28(−) T cells: their role in the age‐associated decline of immune function. Trends Immunol. 30, 306–312. 19540809 10.1016/j.it.2009.03.013 PMC2801888

[acel12349-bib-0093] Wilson VL , Smith RA , Ma S , Cutler RG (1987) Genomic 5‐methyldeoxycytidine decreases with age. J. Biol. Chem. 262, 9948–9951. 3611071

[acel12349-bib-0094] Ziller MJ , Gu H , Müller F , Donaghey J , Tsai LTY , Kohlbacher O , De Jager PL , Rosen ED , Bennett DA , Bernstein BE , Gnirke A , Meissner A (2013) Charting a dynamic DNA methylation landscape of the human genome. Nature 500, 477–481. 23925113 10.1038/nature12433 PMC3821869

